# Identification of exonic regions in DNA sequences using cross-correlation and noise suppression by discrete wavelet transform

**DOI:** 10.1186/1471-2105-12-430

**Published:** 2011-11-03

**Authors:** Omid Abbasi, Ali Rostami, Ghader Karimian

**Affiliations:** 1 School of Engineering-Emerging Technologies, University of Tabriz, Tabriz 5166614761, Iran; 2 Photonics and Nanocrystals Research Lab. (PNRL), Faculty of Electrical and Computer Engineering, University of Tabriz, Tabriz 5166614761, Iran; 3 Faculty of Electrical and Computer Engineering, University of Tabriz, Tabriz 5166614761, Iran

## Abstract

**Background:**

The identification of protein coding regions (exons) in DNA sequences using signal processing techniques is an important component of bioinformatics and biological signal processing. In this paper, a new method is presented for the identification of exonic regions in DNA sequences. This method is based on the cross-correlation technique that can identify periodic regions in DNA sequences.

**Results:**

The method reduces the dependency of window length on identification accuracy. The proposed algorithm is applied to different eukaryotic datasets and the output results are compared with those of other established methods. The proposed method increased the accuracy of exon detection by 4% to 41% relative to the most common digital signal processing methods for exon prediction.

**Conclusions:**

We demonstrated that periodic signals can be estimated using cross-correlation. In addition, discrete wavelet transform (DWT) can minimise noise while maintaining the signal. The proposed algorithm, which combines cross-correlation and DWT, significantly increases the accuracy of exonic region identification.

## Background

**Figure 1:**
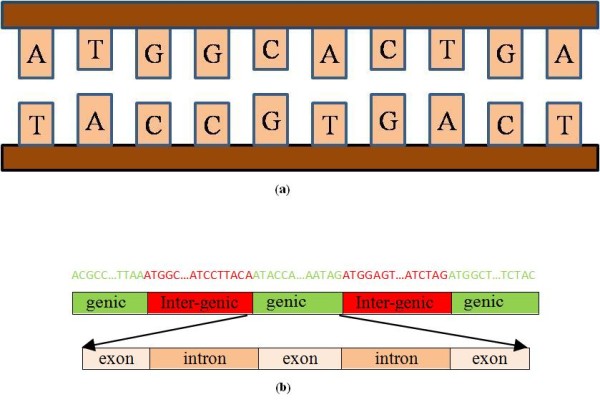
**The structure of DNA sequence**. This figure shows the structure of DNA sequences. (a) Double helix model of DNA. (b) A DNA sequence consists of genes and intergenic regions. The genes of eukaryotes are composed of exons and introns.

When the DNA sequence of a new eukaryotic organism is synthesized, the exonic (protein coding) regions must be distinguished from the introns (see Figure 1 for a schematic of genome arrangement). The protein coding regions of DNA have been observed to exhibit a period-3 property due to the non-uniform codon usage in the translation of codons into amino acids [1]. The aim of this paper is to use this property to identify exonic regions.

Several reasons for the existence of period-3 property have been presented in [2,3] and [4]. Some codons participate more in protein synthesis than others, giving rise to repetitions of a specific type of codon in the genome [4]. For example, the existence of a large number of GCA codons in the exonic regions gives greater repetition of G, C and A nucleotides in the first, second and third codon position, respectively. In other words, the G, C and A nucleotides exhibit period-3 property in the exonic regions.

Gene finding methods based on genetic characteristics, such as promoter, CpG Island, start and stop codon etc., tend to be of insufficient accuracy [5]. The characterization of coding and noncoding regions based on nucleotide statistics inside codons is described by Bernaola et al., who employed a 12-symbol alphabet to identify the borders between coding and noncoding regions [6]. Later, Nicorici and Astola segmented the DNA sequence into coding and noncoding regions using recursive entropic segmentation and stop-codon statistics [7].

The use of signal processing techniques to identify exonic regions based on the period-3 property offers new opportunities for gene finding. Tiawari used Fourier transform spectrum to achieve this goal [8]. In Tiawari's method, the discrete Fourier transform (DFT) energy at a central frequency is calculated for a fixed length window, and the window is slid across the numerical sequence. Vaidyanathan [9] identified protein coding regions using an anti-notch filter which magnified regions with period-3 property. Datta and Asif [10] presented a new algorithm using DFT theory with a Bartlett window. In another signal processing method, Akhtar [11] applied time domain algorithms, average magnitude difference function and time domain periodogram algorithms to identify period-3 property. Some gene finding methods based on digital signal processing (DSP) techniques have been developed but the accuracy of these methods is low and requires improvement.

In this paper, a new algorithm based on cross-correlation theory is presented. We show that the algorithm enhances the accuracy of the identification while reducing noise. The noisy waveform is cross-correlated with a periodic impulse train to provide the estimated signal. Discrete wavelet transform is applied to remove extra frequencies.

The remainder of the paper is organized as follows: in the Methods section, the application of the cross-correlation to obtain the periodic signal plus noise is described, together with the period-3 behaviour detection using cross-correlation theory. The final part of this section details the use of wavelet transform to remove noise. The datasets used are introduced in the Dataset section. Thereafter, evaluation measures are introduced for the measurement and comparison of various methods. Finally, in the Results and Discussion section, the results of the proposed algorithm are compared with those of the most common digital signal processing algorithms for exon prediction, in both time and frequency domains.

## Methods

### Cross-correlation

The discrete nature of DNA and the existence of period-3 behaviour in the exonic regions render it suitable for analysis by signal processing algorithms. We present an algorithm for the identification of the period-3 component based on cross-correlation techniques. The theory of cross-correlation theory is briefly explained below.

Correlation between two waveforms, *x_1_*[*n*] and *x_2_*[*n*], each of length *N*, is defined as [12]:

(1) $$r_{12}=\overset{N-1}{\underset{n=0}{\sum }}x_{1}\left[n\right]x_{2}\left[n\right]$$

To estimate a periodic waveform that is contaminated with noise, this waveform is cross-correlated with an adjustable template waveform; the template waveform is adjusted until the cross-correlation is maximized. The resulting template is an estimate of the signal term of the periodic waveform.

In our approach, a noisy waveform is cross-correlated with a periodic impulse train of period equal to that of the signal.

Let the signal of period *N_p _*points (*N_p _*<*N*) be *s*[*n*] and the noise be *q*[*n*]; therefore, the noisy waveform is *S*[*n*] = *s*[*n*]+*q*[*n*]. Periodic impulse train used for the cross-correlation is denoted *δ*[*n*-*kN_p_*], *k *= 0, 1, 2,..., *N_δ_*, where *N_δ _*is the number of impulses. Then

(2) $$\begin{array}{c} r_{s\delta }\left[j\right]=\frac{1}{N_{\delta }}\overset{N-1}{\underset{n=0}{\sum }}\left(s\left[n\right]+q\left[n\right]\right)\delta \left[n-kN_{p}+j\right],\\ \;\;\;\;\;\;\;\;k=0,1,2,...,N_{\delta } \end{array}$$

Where *j *represents the lag, defined as the number of sampling points by which *δ *is shifted to the left. For *j *= 0, and remembering that *δ*[*n*-*kN_p_*] = 0 for all *n *≠ *kN_p_*

(3) $$\begin{array}{l} r_{s\delta }[0]=\frac{1}{N_{\delta }}(s[0]+q[0]+s[N_{p}]+q[N_{p}]+...\\ \;\;\;\;+s[N_{\delta }N_{p}]+q[N_{\delta }N_{p}])\;\; \end{array}$$

Since the signal is periodic, *s*[*n*+*kN_p_*] = *s*[*n*], and equation (3) becomes

(4) $$\begin{array}{l} r_{s\delta }[0]=\frac{1}{N_{\delta }}(N_{\delta }s[0]+q[0]+q[N_{p}]+...\\ \;\;\;\;\;+q[N_{\delta }N_{p}]) \end{array}$$

or

(5) $$r_{s\delta }\left[0\right]=s\left[0\right]+\frac{1}{N_{\delta }}\overset{N_{\delta }}{\underset{k=0}{\sum }}q\left[kN_{p}\right]$$

As *N_δ _*→ ∞, $\frac{1}{N_{\delta }}\sum_{k=0}^{N_{\delta }}q\left[kN_{p}\right]\to 0$, and therefore *r_sδ _*→ *s*(0).

Now, the periodic impulse train is shifted on the signal by an amount depending on *j*. Thus equation (5) can be written for all *j*'s:

(6) $$r_{s\delta }\left[j\right]=s\left[j\right]+\frac{1}{N_{\delta }}\overset{N_{\delta }}{\underset{k=0}{\sum }}q\left[kN_{p}+j\right]$$

From equation (6), it can be concluded that

(7) $$r_{s\delta }\left[j\right]=s\left[0\right],\;s\left[1\right],\;...,\;s\left[N-1\right]\;\;j=0,1,2,...,N-1$$

from which the periodic signal without noise can be extracted [12].

### Identification of exonic regions

In this section, a new algorithm using the cross-correlation is proposed for the identification of exonic regions. The algorithm proceeds via the following steps:

1. DNA sequences are converted into numerical sequences.

2. FIR filter is applied to the numerical sequences representing DNA sequences.

3. Cross-correlation is applied to the filtered numerical sequences.

4. The noise effect is removed using discrete wavelet transform.

**Figure 2:**
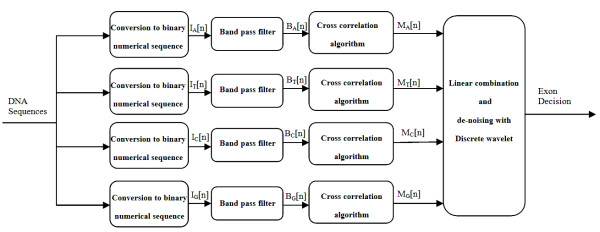
**Block diagram of the proposed algorithm**. This figure shows the block diagram of the proposed algorithm designed to identify protein coding regions.

Figure 2 represents these steps as a block diagram. Each step is explained in detail below:

#### 1. Numerical conversion of the DNA sequences

To apply DSP techniques to the DNA sequence to find nucleotide regions exhibiting period-3 behaviour, the DNA sequence is first mapped onto the numerical sequence. The simplest conversion method maps four numerical sequences *I_A_*[*n*], *I_T_*[*n*], *I_C_*[*n*] and *I_G_*[*n*] from DNA sequences in binary format. In this mapping, the presence or absence of the respective nucleotides at the *n*th position is represented by '1' and '0', respectively. For example, given a section of DNA sequence ATCCGATATTC, the binary sequence of the nucleotide A, denoted *I_A_*[*n*], is [10000101000]. The binary sequences for the other three nucleotides T, C and G are found similarly [13].

#### 2. Applying FIR filter to the numerical sequences

After mapping the DNA sequence onto its binary numerical sequence, the binary sequence is passed through a Hamming window based FIR filter of order 8 with central frequency set to 2π/3, to emphasize period-3 property in the exonic regions. Lack of distortions in FIR filters is one reason for their preferred use over IIR filters in medical applications [12].

#### 3. Applying cross-correlation theory to the numerical sequences

Most previous methods have used a window of fixed length to find the regions in DNA sequences exhibiting period-3 property. In such methods, the window length directly affects the accuracy of the identification. Typically, an appropriate window length is considered to lie within the range 240-351 (window lengths are multiples of three to reflect the codon structure). Short length windows increase noise, while long length windows tend to miss short exonic regions.

In our proposed method, the cross-correlation between the numerical DNA sequence and an impulse train of periodicity 3 (*N_p _*= 3) is calculated to identify regions in the DNA sequence with period-3 behaviour. The length of the impulse train is set at 270. The impulse train signal length plays the same role as the window length in previous approaches. Following the cross-correlation calculation, the impulse train slides across the numerical sequence by an amount *j*. The impulse train with periodicity of three considered as *δ*[*n*-3*k*] and B_A_[n], B_T_[n], B_C_[n] and B_G_[n] are the FIR filter outputs for the input I_A_[n], I_T_[n], I_C_[n] and I_G_[n] sequences respectively. Then,

(8) $$M_{A}=\overset{N-1}{\underset{n=0}{\sum }}B_{A}\left[n\right]\delta \left[n-3k\right]$$

(9) $$M_{T}=\overset{N-1}{\underset{n=0}{\sum }}B_{T}\left[n\right]\delta \left[n-3k\right]$$

(10) $$M_{C}=\overset{N-1}{\underset{n=0}{\sum }}B_{C}\left[n\right]\delta \left[n-3k\right]$$

(11) $$M_{G}=\overset{N-1}{\underset{n=0}{\sum }}B_{G}\left[n\right]\delta \left[n-3k\right]$$

Different energy levels of the period-3 components exist in binary sequences M_A_, M_T_, M_C _and M_G_. Thus, the output energy spectrum is the combination of the four separate outputs

(12) $$M=M_{A}+M_{T}+M_{C}+M_{G}$$

In this energy spectrum, a peak corresponds to the presence of a period-3 component on that region, implying that the region is exonic.

#### 4. Decreasing the noise using discrete wavelet transform

Decreasing noise increases the accuracy of exonic region identification. As seen from equation (6), a small window size, required for the detection of small exons, will not diminish noise sufficiently. Hence we apply discrete wavelet transform (DWT) to decrease the noise in the output spectrum.

DWT has been used for de-noising in various signal processing applications. In protein coding region detection, Haar wavelet has previously been employed for noise suppression [14]. Our proposed algorithm uses Dmey wavelet to remove noise and thereby increase the accuracy of the exonic region identification.

**Figure 3:**
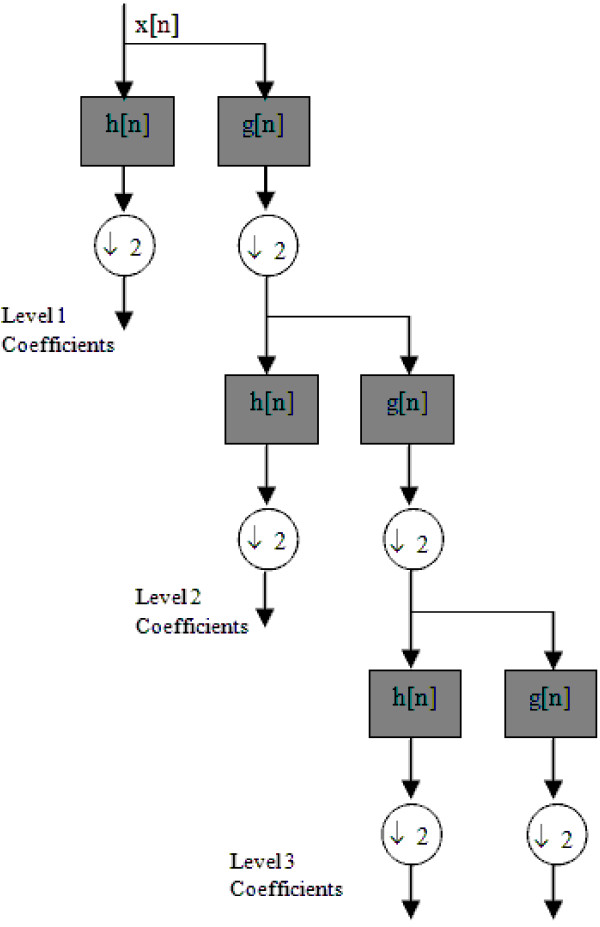
**DWT decomposition**. This figure shows the schematic of DWT decomposition at three levels. The low pass and high pass half-band filters are denoted *g*[*n*] and *h*[*n*] respectively.

To this end, by down-sampling the output of low pass and high pass filters, samples are divided into two signals; high frequency samples (detail signals) and low frequency samples (approximation signals), each embracing half the number of samples as the original signal. Figure 3 shows this procedure operating over three levels.

The signal *x*[*n*] is passed first through the high pass filter, *h*[*n*], then through the low pass filter, *g*[*n*] [14,15].

(13) $$s_{high}\left[k\right]=\underset{n}{\sum }x\left[n\right].h\left[2k-n\right]$$

(14) $$s_{low}\left[k\right]=\underset{n}{\sum }x\left[n\right].g\left[2k-n\right]$$

**Figure 4:**
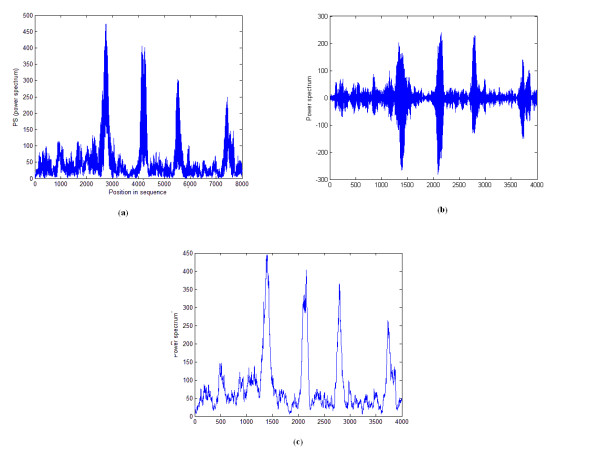
**Applying DWT to the proposed algorithm**. This figure shows the results of applying DWT to the proposed algorithm for the sequence F56F11.4. (a) The output power spectrum of the proposed algorithm before DWT is applied. (b) High frequency components of level 1 DWT decomposition (detail signal). (c) Low frequency components of level 1 DWT decomposition (approximation signal).

Approximation and detail signals for the output power spectrum of the sequence F56F11.4 (GenBank access number AF099922) at positions 7021-15020 are shown in Figures 4b and 4c. By removing the detail signal and considering only the approximation signal, the extra frequencies are removed and the output power spectrum is smoothed. Therefore, the noise effect is decreased, while the accuracy of the identification is enhanced.

### Datasets

Standard datasets are used to compare the efficacy of different algorithms at identifying exonic regions. Exon and intron positions in these databases are available and when DSP methods detect the position of exons, these positions are compared with real positions. The proposed algorithm is first applied to chromosome III of *Caenorhabditis elegans *[NCBI Reference Sequence: NC_003281.8], containing a total of 13783681 nucleotides with 8172 coding regions, and the results are compared with those of other popular methods. The results of the proposed algorithm for the sequence F56F11.4 of *C. elegans *(comprising 8,000 nucleotides) are separately presented. This sequence has five exonic regions at positions 928-1039, 2528-2857, 4114-4377, 5465-5644, and 7255-7605. Also analysed in this paper are the BG570 [16] and HMR190 [17] datasets. BG570 is a genomic test dataset of 570 single gene vertebrate sequences prepared by Burset and Guigo [16]. HMR195 comprises 195 single-gene human, mouse, and rat sequences selected in 2001 by Rogic et al. [17] to test and evaluate the performance of gene structure prediction algorithms.

### Evaluation Measures

To accurately compare different methods, the evaluation is performed at the nucleotide level. In the identification of exonic regions using DSP techniques, some parameters are defined by changing the threshold level in the output spectrum. Those parameters which make the comparison possible are defined in this section. In the identification step, the number of nucleotides correctly predicted as exons is denoted true positive (represented by TP), while the number of nucleotides correctly predicted as introns is denoted true negative (represented by TN). Similarly, the number of intron nucleotides predicted as exon nucleotide is the false positive (FP) value, while the number of exon nucleotides predicted as intron nucleotides is the false negative (FN) value. From these four defined quantities, the sensitivity and specificity parameters are determined as follows [16]:

(15) $$S_{n}=\frac{TP}{TP+FN}$$

(16) $$S_{p}=\frac{TP}{TP+FP}$$

The sensitivity *S_n _*is the proportion of exon nucleotides that have been correctly predicted as exons, and the specificity *S_p _*is the proportion of predicted exon nucleotides that actually exist in the exonic regions. These parameters alone are not suitable for evaluation because at high sensitivity, the specificity is low and vice versa. Therefore, another measure known as the approximate correlation (AC) has been defined. This parameter combines sensitivity and specificity as shown [16].

(17) $$\begin{array}{l} ACP=\frac{1}{4}*(\frac{TP}{TP+FN}+\frac{TP}{TP+FP}+\\ \;\;\;\frac{TN}{TN+FN}+\frac{TN}{TN+FP}) \end{array}$$

(18) AC=(ACP-0.5)*2

In applying DSP techniques to gene searching, other parameters have been described. A most popular evaluation measure is the Receiver Operating Characteristic (ROC) curve. By selecting different threshold levels, different values of TP for a given FP are calculated at each threshold and the ROC curve is constructed from the various TPs and their corresponding FPs. The area under the ROC curve (AUC) is used as an evaluation measure; the greater the AUC, the higher the accuracy of the gene finding algorithm [18]. Another means by which to compare identification accuracy between methods is the calculation of specificity for different sensibilities. Since the majority of genomes comprise intronic and intergenic regions, the calculation of FP can provide a useful comparison measure [19].

### Threshold Selection Method

To discriminate between coding and noncoding regions, a threshold is imposed on the output power spectrum. The selection of a proper threshold can optimise the accuracy of the identification; however, the calculation of an optimum threshold value itself raises problems [20]. Therefore, in this paper, the sensitivity, specificity and approximate correlation measures are defined by changing the threshold level, to accurately compare different methods. In this section, we discuss implementation of the threshold selection.

To select an appropriate threshold, the method of Kwan et al. [21] is used. The mean and standard deviation of the period-3 values determined from a training set of exon and intron sequences are used to calculate the threshold level *T*, defined as:

(19) $$T=\frac{sdP_{3e}*meanP_{3i}+sdP_{3i}*meanP_{3e}}{sdP_{3e}+sdP_{3i}}$$

where *meanP_3e _*and *sdP_3e _*represent respectively the mean and standard deviation of the period-3 values obtained from the exon sequences of a training set, and *meanP_3i _*and *sdP_3i _*represent respectively the mean and standard deviation of the period-3 values obtained from the intron sequences of the same training set.

**Figure 5:**
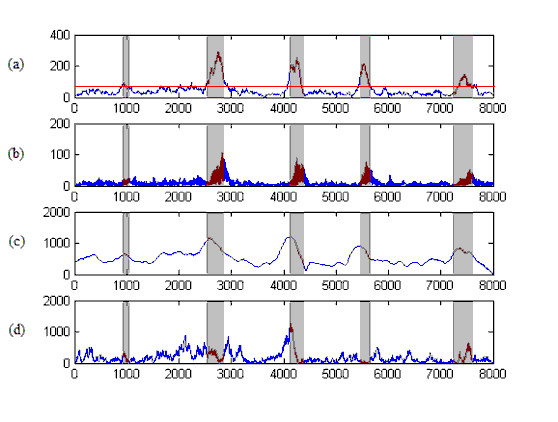
**The identification of exonic regions on the gene sequence F56F11.4**. The results of exonic region identification on the sequence F56F11.4 (8,000 bp) are plotted for different methods. (a) Cross-correlation (proposed), (b) AN filter, (c) TDP and (d) DFT methods. The shadowed regions are exonic regions that must be identified.

The 1000 multi exon genes from chromosome III of *C. elegans *provide data for training. The calculated threshold level is 61. This threshold level was applied to the F56F11.4 gene in chromosome III of *C. elegans *as shown in Figure 5.a. Clearly, at this threshold, all five regions are correctly identified as coding regions. However, there also exist small non-coding regions around position 2000 which are misidentified as coding regions. Since the characteristics of the DNA sequence can change significantly at different positions, even within the same dataset, a static threshold may yield incorrect identifications at some positions. Therefore, an adaptive threshold selection algorithm such as that described in [22] is required for exon prediction. In Tables (1), (2), (3), and (4) our proposed algorithm is compared with other algorithms over a range of thresholds.

## Results and Discussion

**Figure 6:**
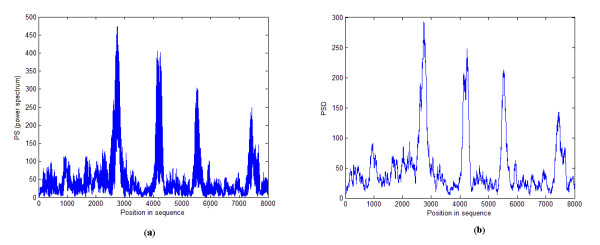
**Results of applying DWT to the proposed algorithm for the gene sequence F56F11.4**. This figure shows the results of applying DWT to the proposed algorithm for the sequence F56F11.4. (a) The output power spectrum of the proposed algorithm before DWT is applied. (b) The output power spectrum of the proposed algorithm following DWT processing.

**Figure 7:**
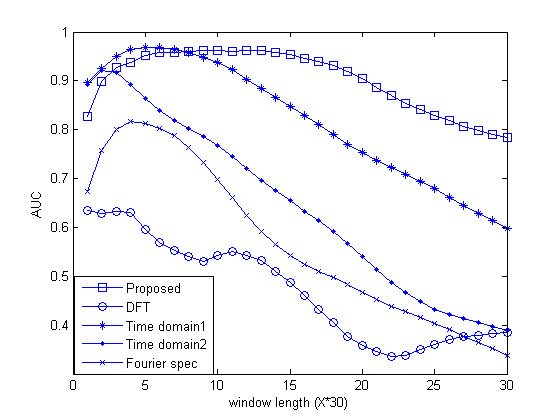
**The effect of window length**. Area under the ROC curves generated by different methods for the gene sequence F56F11.4 is plotted. In our algorithm, the accuracy of identification is fixed for window lengths between 150 and 510 bp, but changing the window length affects the identification accuracy of the other methods.

In this section, the results of the proposed algorithm are compared with those of established methods, namely, Average Magnitude Difference Function (AMDF), Time Domain Periodogram (TDP) [11], Anti-Notch filter (AN filter) [9], Fourier Transform Spectrum (DFT) [8] and Asif [10]. As mentioned in the previous section, to evaluate and compare the results, measures such as the area under the ROC curve, the specificity and the number of false negatives in a particular sensitivity are computed. The approximate correlation measure for different threshold levels is also calculated. Our proposed algorithm is first applied to the gene sequence F56F11.4. In Figure 5a, the output power spectrum computed by equation (12) is displayed. The output power spectrum of the AN filter, TDP and DFT methods is shown in Figures 5.b, c and 5d respectively. Exonic regions that should be identified in this figure are marked as shaded regions. It should be noted that Figure 5.a is the output of equation (12) after de-noising with DWT, as shown in Figure 6.b. The strongest feature of our proposed algorithm is the noise reduction. Not only is the noise reduced by increasing the window length, but the small length exonic regions can be identified. Unlike the established methods, the accuracy of identification does not decrease by changing the window length up to a specific value. Figure 7 shows the effect of changing window length on area under the ROC curve for the F56F11.4 sequence. According to this curve, the identification accuracy of our algorithm is fixed for window lengths ranging from 150 to 510 bp, whereas that of the other tested methods depends on window length. The window length varies according to gene length. The decreasing noise effect and magnification of the period-3 component under FIR filtering causes the peaks to coincide with exon positions and enables detection of small exons, such as the first exon in F56F11.4 (shown in Figure 5.a).

In Table 1, the approximate correlation and specificity for specified sensitivities are presented for our proposed method and for the other tested methods (using gene sequence F56F11.4). We observe that our algorithm yields the highest value of both parameters.

**Table 1:** Comparison of different methods using the sequence F56F11.4.

Method	*S_n_*	*S_p_*	AC
DFT	0.80	0.17	0.08
AN filter	0.80	0.23	0.25
Asif	0.80	0.18	0.12
AMDF	0.80	0.20	0.19
TDP	0.80	0.49	0.55
Cross-correlation (Proposed)	**0.80**	**0.82**	**0.78**

Table 1 shows the approximate correlation (AC) measure and specificity (*S_p_*) at the same sensitivity (*S_n_*) for our proposed method and for other methods using the sequence F56F11.4.

The proposed algorithm is then applied to chromosome III of *Caenorhabditis elegans *[NCBI Reference Sequence: NC_003281.8], comprising 13783681 nucleotides with 8172 coding regions, and the results are again compared with the outputs of other popular methods. Different evaluation measures for the proposed algorithm, AN filter and TDP methods are shown in Table 2. Clearly, the proposed method outperforms AN filter and TDP methods. It achieves a larger area under the ROC curve, fewer false positives and higher specificities and approximate correlation compared with AN filtering and TDP. By way of illustration, at a sensitivity of 20% the false positive output of our algorithm is 134 bp compared with 157 bp and 196 bp for AN filtering and TDP, respectively. In addition, our proposed method exhibits relative improvements of 3% and 5% respectively over AN filter and TDP methods in the approximate correlation measure.

**Table 2:** Evaluation of different methods using chromosome III of *C. elegans*

		*S_n_*								
		
		%20			%40			%60		
		
Methods	AUC	FP	*Sp*	AC	FP	*Sp*	AC	FP	*Sp*	AC
AN filter	0.6471	157	71	0.17	372	66.3	0.21	727	60	0.20
TDP	0.6115	196	70	0.15	436	65	0.18	796	59	0.19
Cross-correlation (proposed)	**0.6891**	**134**	**76.5**	**0.20**	**302**	**70.9**	**0.25**	**610**	**61**	**0.26**

Table 2 shows the area under the ROC curve (AUC), the number of false positives (FP), the approximate correlation (AC) and specificity (*S_p_*) at the same sensitivity (*S_n_*) for our proposed method and for other methods using the chromosome III of *C. elegans*.

The proposed algorithm was finally applied to the HMR195 and the BG570 datasets. The output results are shown in Table 3. With regard to the HMR195 dataset, our algorithm outputs the least number of nucleotides incorrectly identified as exons. At a sensitivity of 30%, the number of false positives in the cross-correlation method is improved by a factor of 1.24 relative to the next-best performing method, Asif. Our proposed algorithm shows relative improvements of 21%, 8.3%, 24%, 18% and 4% over the DFT, AN filter, Asif, AMDF and TDP methods respectively, in terms of the area under the ROC curve. Similar superiority of our proposed algorithm is apparent for the BG570 dataset.

**Table 3:** Evaluation of different methods using HMR195 and BG570 genomic datasets.

	BG570							HMR195						
	
		*S_n_*							*S_n_*					
			
		%10		%30		%50			%10		%30		%50	
			
Methods	AUC	FP	*S_p_*	FP	*S_p_*	FP	*S_p_*	AUC	FP	*S_p_*	FP	*S_p_*	FP	*S_p_*
DFT	0.6540	279	45.8	767	43.3	1412	34.3	0.6782	438	51.5	1184	45	2064	41.7
AN filter	0.6765	121	55	499	49.7	1103	36.7	0.7615	151	64.4	526	57.4	1217	51.1
Asif	0.5748	140	34.2	330	31.7	554	29.1	0.6261	214	47.1	473	44.6	787	39.9
AMDF	0.6600	340	40.8	770	39.4	1309	35.3	0.6980	410	47.9	1010	46.8	1821	43.3
TDP	0.7560	160	62	408	56	805	49.4	0.7850	262	64.8	627	60.4	1128	56
Cross-correlation (proposed)	**0.8143**	**81**	**75.5**	**244**	**69**	**547**	**61**	**0.8250**	**124**	**71**	**382**	**67**	**841**	**59**

Table 3 shows the area under the ROC curve (AUC), the number of false positives (FP), the approximate correlation (AC) and specificity (*S_p_*) at a given sensitivity (*S_n_*) for the proposed method and for other methods using the BG570 and HMR195 datasets.

**Figure 8:**
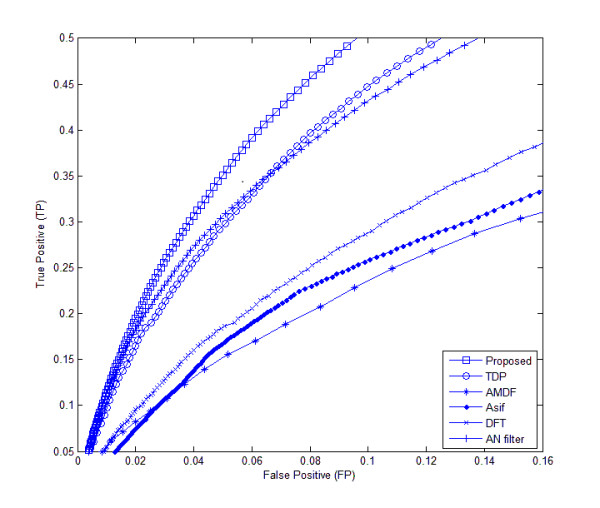
**ROC curves of different methods for the HMR195 genomic dataset**. The ROC curves of different methods are plotted for the HMR195 dataset.

**Figure 9:**
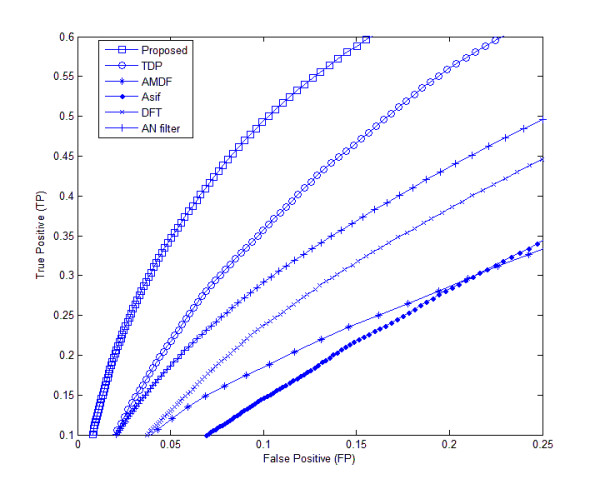
**ROC curves of different methods for the BG570 genomic dataset**. The ROC curves of different methods are plotted for the BG570 dataset.

Table 4 shows the AC measure of our proposed method in addition to the other tested methods. At a sensitivity of 80%, the AC measure for the proposed method is 40% in the BG570 database, while that of TDP (yielding the highest AC of the established methods) is 31%. Finally, from Figures 8 and 9, illustrating the ROC's of the proposed and other methods, it is obvious that the proposed method's area under curve in both datasets is the highest of all the tested methods. This implies that our proposed algorithm is superior to the other methods at identifying exonic gene regions.

**Table 4:** Approximate correlation measures for HMR195 and BG570 genomic datasets

	BG570			HMR195		
	
method	*S_n_*	*S_p_*	AC	*S_n_*	*S_p_*	AC
DFT	0.80	0.28	0.18	0.80	0.31	0.18
AN filter	0.80	0.26	0.17	0.80	0.39	0.32
Asif	0.80	0.25	010	0.80	0.30	0.15
AMDF	0.80	0.29	0.20	0.80	0.37	0.27
TDP	0.80	0.37	0.31	0.80	0.44	0.38
Cross-correlation (Proposed)	**0.80**	**0.43**	**0.40**	**0.80**	**0.47**	**0.45**

Table 4 shows the approximate correlation (AC) measure for our proposed method and for other methods for a given sensitivity (*S_n_*) and specificity (*S_p_*).

## Conclusions

This paper presents a new algorithm based on cross-correlation theory, designed to increase the accuracy of exonic region identification. The FIR filter makes it easier to identify the exonic regions. The main advantage of the proposed method is its reduced dependency on the window length as a result of the decreasing noise effect. The ability to detect small exonic regions is another advantage of this algorithm. The final step of the algorithm utilizes the discrete wavelet transform to reduce noise. Compared with established time and frequency domain methods, the proposed algorithm yields improvements ranging from 4% to 41% in terms of the area under the ROC curve for the HMR195 and BG570 datasets. Our proposed method also minimises the number of nucleotides incorrectly predicted as exonic. This decrease in the number of false positives is responsible for the increase in specificity; for example, at a sensitivity of 30%, our proposed algorithm yielded 15% to 85% improvement in specificity over other tested methods. As can be seen from Tables 3 and 4, our algorithm confers significant improvement on the accuracy of exonic region identification.

## Authors' contributions

This work is carried out under the close guidance of AR and GK, who conceived of the study, and who participated in its design and coordination. OA implemented the method. The manuscript was written by OA and edited by AR and GK. All authors have read and approved the final manuscript.
